# Multiple delayed-onset metachronous ileal stenoses after transcatheter arterial embolization using *N*-butyl-2-cyanoacrylate for upper gastrointestinal bleeding: factors of complication and importance of plain CT evaluations

**DOI:** 10.1093/bjrcr/uaae042

**Published:** 2024-11-04

**Authors:** Zenjiro Sekikawa, Hiroyuki Kamide, Yusuke Kobayashi, Miki Terauchi

**Affiliations:** Department of Diagnostic Radiology, Yokohama City University Medical Center, Minami-ku, Yokohama 232-0024, Japan; Department of Diagnostic Radiology, Yokohama City University Medical Center, Minami-ku, Yokohama 232-0024, Japan; Department of Diagnostic Radiology, Yokohama City University Medical Center, Minami-ku, Yokohama 232-0024, Japan; Department of Radiology, Japan Organization of Occupational Health and Safety (JOHAS), Yokohama Rosai Hospital, Kohoku-ku, Yokohama 222-0036, Japan

**Keywords:** *N*-butyl-2-cyanoacrylate, NBCA, transcatheter arterial embolization, TAE, plain CT, complication, metachronous, delayed-onset, ileal stenosis, dilution

## Abstract

A 63-year-old man underwent transcatheter arterial embolization (TAE) using a mixture of *N*-butyl-2-cyanoacrylate (NBCA) and iodized oil to treat acute gastrointestinal (GI) bleeding. The procedure was initially successful; however, the patient developed ileus >1 month later and subsequently underwent several surgeries to treat the multiple metachronous ileal stenoses. The flux of a small amount of off-target glue was the primary cause of these complications. As the patient had few symptoms in the first month post-TAE, however, affirming the diagnosis took time. A detailed review of plain CT scans was a decisive factor in achieving the final diagnosis. This case demonstrates that TAE using an NBCA–iodized oil mixture effectively treats acute GI bleeding. However, a complication such as off-target embolization is likelier to occur because of a combination of certain factors such as vascular anatomy, complexity of the procedure, and NBCA dilution. Close observation using plain CT should be performed for the identification of off-target embolization occurrence even in cases of successful TAE.

## Background

Transcatheter arterial embolization (TAE) using a mixture of *N*-butyl-2-cyanoacrylate (NBCA) and iodized oil is considered safe and effective for achieving immediate haemostasis in the treatment of acute gastrointestinal (GI) bleeding. Although several studies have highlighted the benefits of this successful treatment and its associated complications, few have analysed and evaluated the causes of complications in detail. Off-target embolization, a major cause of complications, is often overlooked when symptoms are absent or mild. Despite being a major complication, intestinal strictures typically subside after minor treatments or short-term observation. Contrast-enhanced CT is routinely performed post-TAE to confirm haemostasis, and plain CT is often performed simultaneously. However, clinicians tend to focus on evaluating the contrast-enhanced CT image, potentially leading to an insufficient assessment of plain CT scans. Herein, we present a case of multiple metachronous ileal stenoses that developed >1 month after TAE using NBCA to treat acute upper GI haemorrhage; plain CT enabled the detection of an initially unnoticed off-target embolization. The complication in this case was likelier to occur because of a combination of certain factors such as vascular anatomy, complexity of the procedure, and NBCA dilution. In addition to contrast-enhanced CT, this case highlights the importance of plain CT evaluations, even in cases in which the TAE procedure seems initially successful, for detecting any off-target embolizations.

## Case report

**Figure 1.**
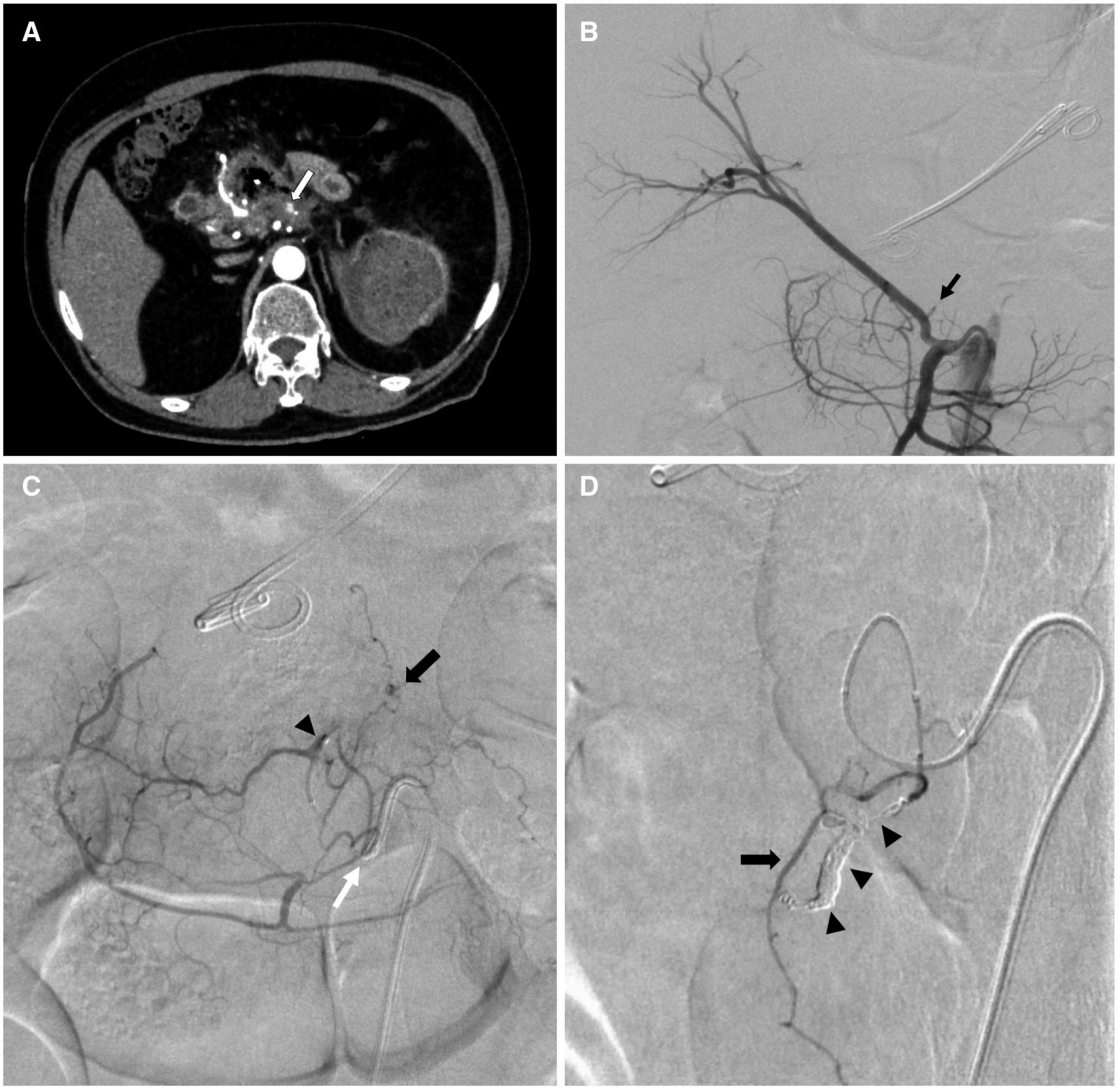
CT and angiographic images of the first transcatheter arterial embolization (TAE). (A) Contrast-enhanced CT showing a minute pseudoaneurysm around the abscess (arrow). (B) Angiogram of the superior mesenteric artery showing replaced right hepatic artery and the orifice of its first small branch (arrow). (C) Angiogram of the first branch of the replaced right hepatic artery shown in panel B (white arrow: orifice of the artery) demonstrating a small pseudoaneurysm (arrow) in the far distal portion of the left branch (arrowhead). (D) Angiogram of the distal part of the left branch indicated in panel C acquired using a 1.5-F microcatheter. A branch of the pancreaticoduodenal artery was occluded using microcoils to prevent off-target embolizations (arrowheads). The injection of the *N*-butyl-2-cyanoacrylate–lipiodol mixture resulted in minimal flow towards the small duodenal branch (arrow).

A 63-year-old man underwent a distal pancreaticoduodenectomy to treat a neuroendocrine tumour. Three months later, the patient presented to the hospital with a peripancreatic abscess, and transgastric drainage was performed. One month later, the patient experienced haematemesis-induced shock. Contrast-enhanced CT revealed a small pseudoaneurysm around the abscess (Figure 1A) and the replacement of the right hepatic artery with the superior mesenteric artery (SMA; not depicted in figures). The coeliac artery and SMA were navigated by inserting a guiding sheath (Parent Plus 45; MEDIKIT, Tokyo, Japan) into the right femoral artery. Subsequently, we obtained a gastroduodenal artery (GDA) arteriogram using a 2.6-F microcatheter (Carry Leon; UTM, Aichi, Japan). No pseudoaneurysms or extravasations were observed. After the CT observations, a small pancreatic branch originating from the replaced right hepatic artery (Figure 1B) was selected using a 2.9-F tip-steerable catheter (Leonis Mova; Sumitomo Bakelite, Tokyo, Japan), and the angiogram showed a pseudoaneurysm at the distal point of the left arterial branch (Figure 1C). A triple coaxial system using a 1.5-F microcatheter (Carry Leon) was established; however, catheterization of the target artery was impossible.

NBCA was selected after initial microcoil deployment to confirm solid haemostasis and avoid off-target embolization. The pancreaticoduodenal artery branching near the target artery was occluded using a microcoil (Target XL; Stryker, Fremont, CA, USA). However, because of its extremely small and sharply curved vessels, the small duodenal branch could not be occluded. A mixture of 0.3 mL of NBCA and iodized oil (Lipiodol; Guerbet Japan, Tokyo, Japan) in a ratio of 1:8 was injected following the administration of 5% dextrose, and haemostasis of the target artery was confirmed. However, some of the mixture flowed into the smaller duodenal branches (Figure 1D).

**Figure 2.**
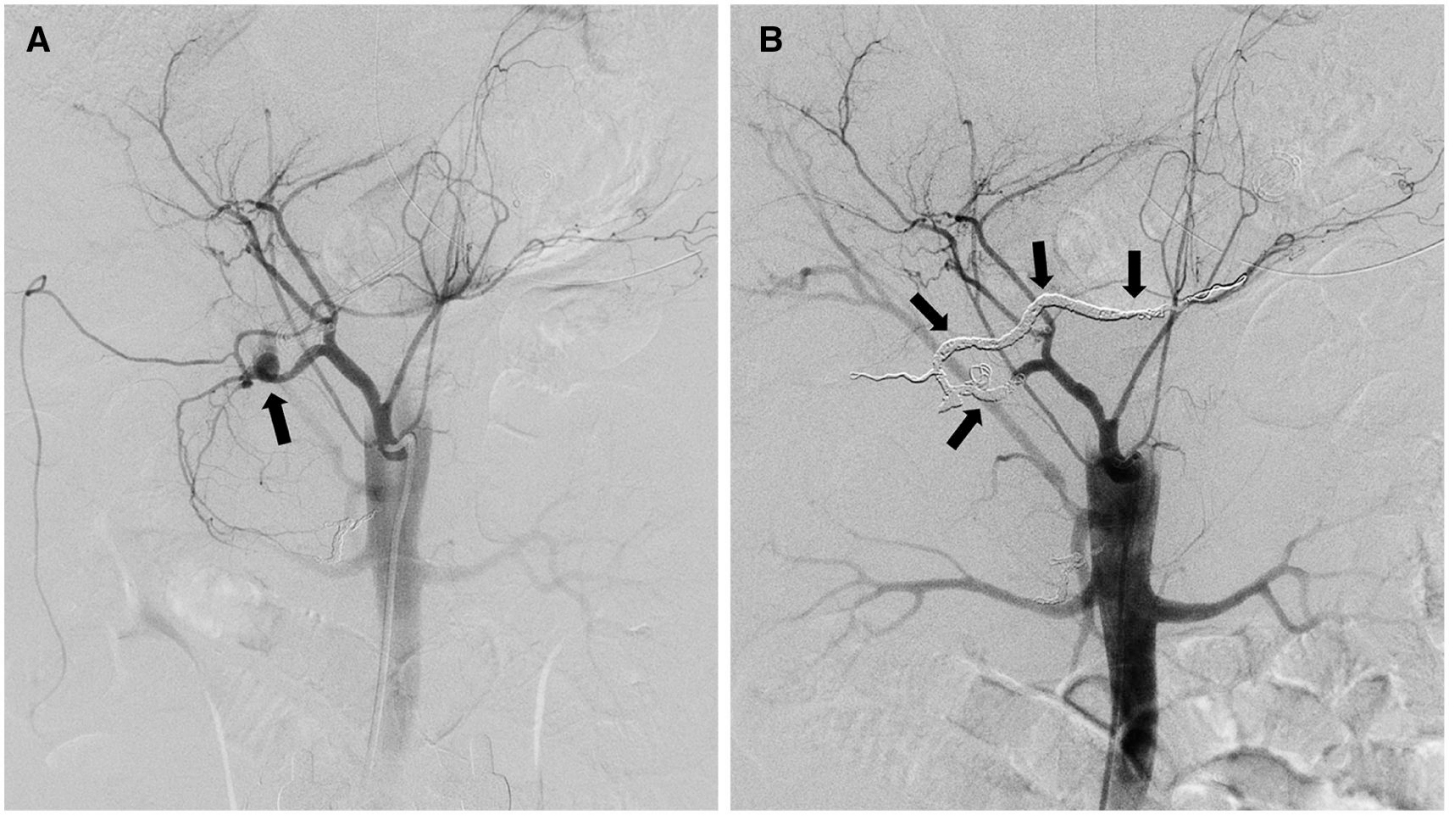
Angiographic images of the second transcatheter arterial embolization. (A) Angiogram of the coeliac trunk depicting a pseudoaneurysm (arrow) in the distal portion of the gastroduodenal artery (GDA). (B) After coil embolization, microcoils were positioned from the distal region of the right gastroepiploic artery to the proximal area of the GDA, effectively encompassing all inflammatory regions (arrows).

The patient complained of flank pain immediately after injection, which gradually subsided over time. However, the patient developed haemorrhagic shock 4 days later. Contrast-enhanced CT and angiography revealed a newly developed pseudoaneurysm in the GDA (Figure 2A). Coil embolization (Ruby POD; Penumbra, Alameda, CA, USA) was performed using a 2.6-F microcatheter to cover the pseudoaneurysm and entire inflammatory region: from the distal right gastroepiploic artery to the proximal GDA (Figure 2B). Complete haemostasis was verified using coeliac and SMA angiography.

**Figure 3.**
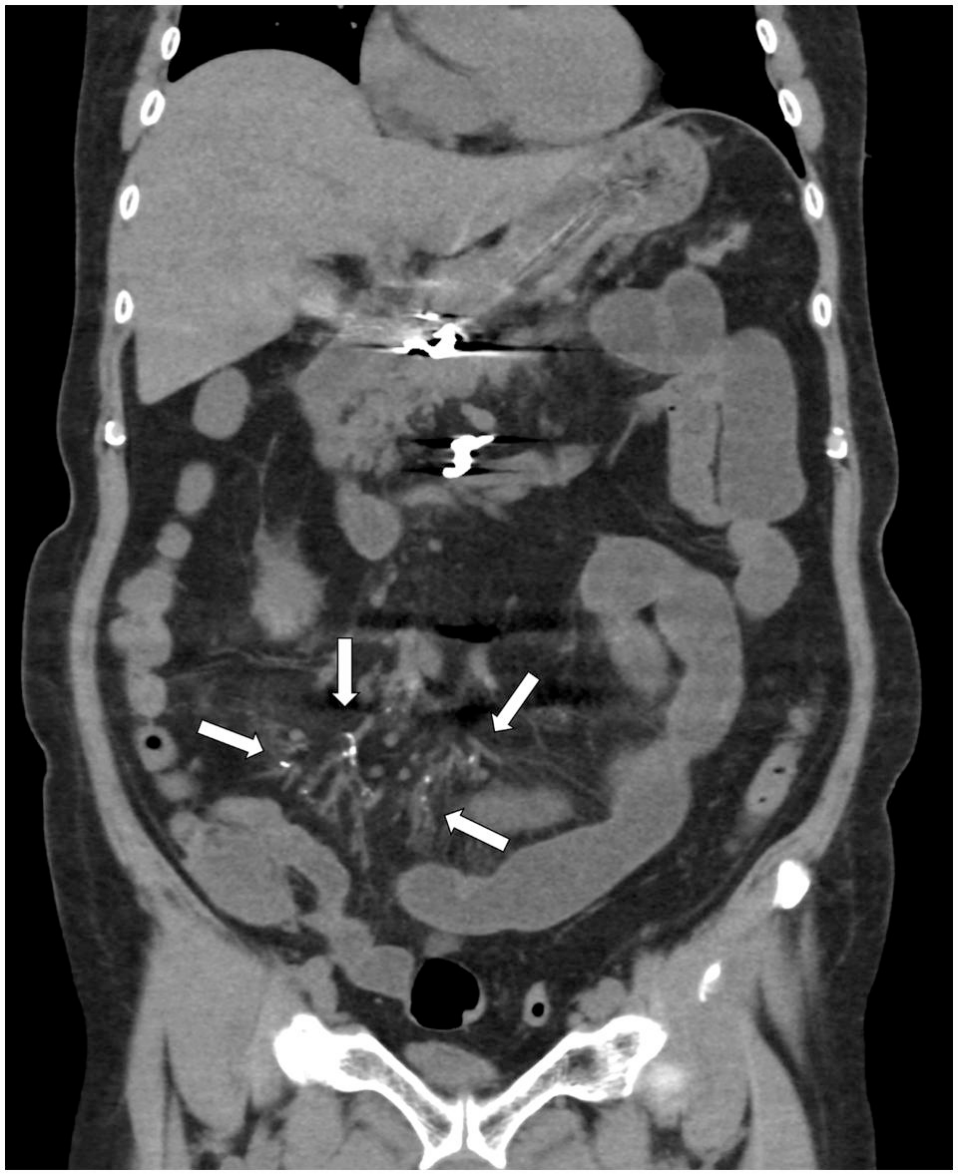
Coronal image of a plain CT scan following transcatheter arterial embolization displaying dot-like high-density areas (arrows) along the distal ileal arteries.

**Figure 4.**
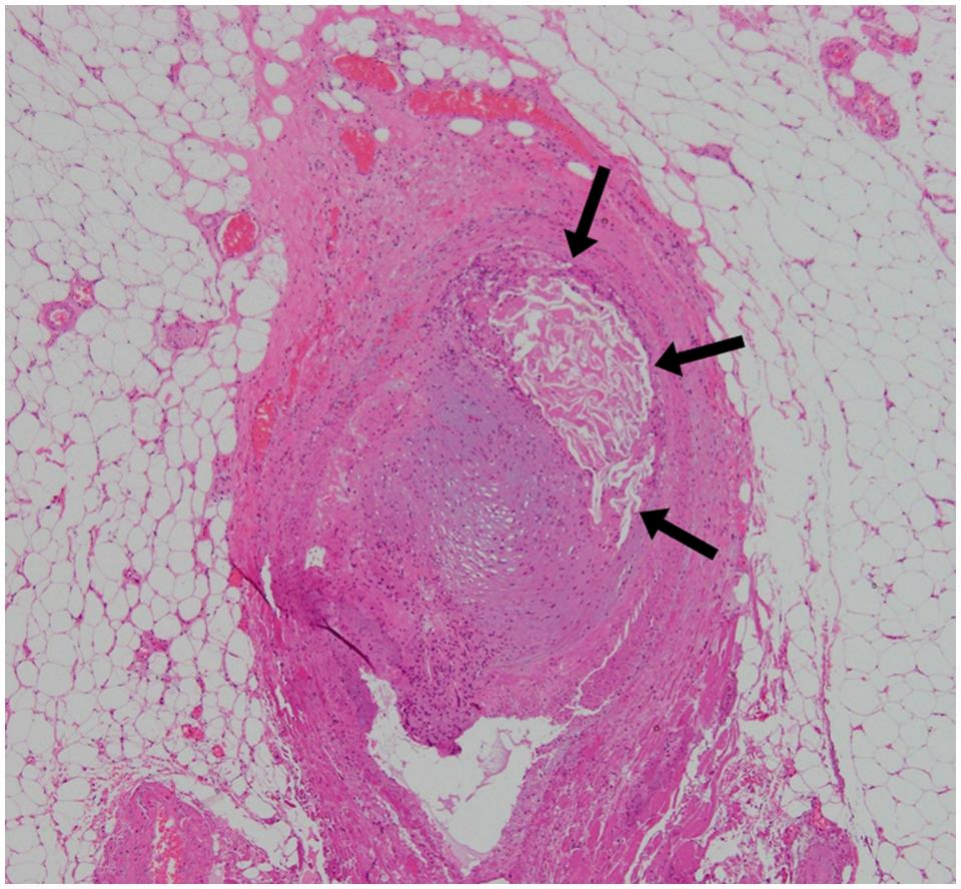
Strongly magnified pathology image of the haematoxylin-eosin staining of the resected intestine. A foreign body, believed to be *N*-butyl-2-cyanoacrylate glue, was observed within a small artery in the sub-peritoneal fat layer (arrows). A cluster of macrophages and fibroblasts was observed in the surrounding area. These findings were consistent with those of ischaemic enteritis caused by foreign bodies.

The patient’s postoperative course was uneventful, and he resumed regular eating habits 1 month later. However, the patient subsequently developed fever and ileus, which were attributed to stenosis of the terminal ileum. Although the reason for this was unclear, the patient tested positive for cytomegalovirus antigen and was treated for cytomegalovirus enteritis. Five CT scans (both plain and contrast-enhanced) were performed post-TAE to confirm haemostasis and check for pseudoaneurysms and abscesses. Considering the possibility of off-target embolization, the previous plain CT scans were carefully reviewed, and multiple dotted high-density areas in the branches of the ileal artery were found (Figure 3). Partial ileal resection was performed, and the histopathological findings revealed ischaemic enteritis caused by foreign bodies (Figure 4).

The patient experienced a new ileus episode 2 months postoperatively (3 months post-TAE). Ileocecal resection was performed to treat the newly developed recurrent stenosis in another part of the ileum. The patient was discharged 2 weeks later without postoperative complications and reported no major GI problems for >1 year after discharge.

## Discussion

TAE, using a mixture of NBCA and iodized oil, is widely acknowledged as a safe, efficient, and minimally invasive method for the treatment of GI bleeding. Several studies have highlighted its efficacy, particularly in the upper GI tract.^1–5^ The technical success rate of TAE with NBCA exceeds 98% for both upper and lower GI bleeding,^1^^,^^4^ and the clinical success rate is higher for the former than the latter (88.0% and 78.0%, respectively).^1^ NBCA has many advantages over other embolic agents, such as coils and gelatin particles; however, its complications are unfrequently assessed. Some studies have demonstrated GI ischaemic complications following TAE using NBCA,^4^^,^^6–8^ including one case that required gastrojejunostomy following non-super-selective embolization using NBCA glue.^7^ NBCA-related bowel complications tended to be mild and occur immediately after TAE. One case report described a colonic stricture that developed 6 months post-TAE using NBCA.^9^

A contrast-enhanced CT is routinely performed after TAE to confirm haemostasis. Plain CT scans are often performed simultaneously with contrast-enhanced CT. However, clinicians tend to focus on evaluating contrast-enhanced CT images, potentially leading to an insufficient assessment of plain CT scans. In the present case, failure to evaluate the plain CT scan delayed diagnosis. Although the flux of the non-target NBCA–lipiodol mixture was considered negligible, an appropriate evaluation of the plain CT scan should have been performed when the patient developed symptoms of enteritis.

**Figure 5.**
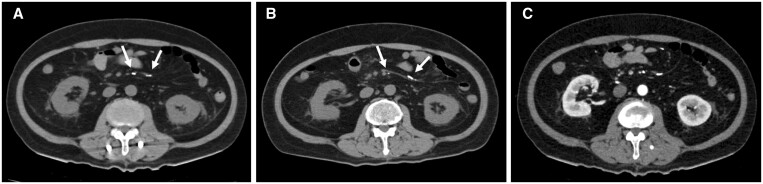
Axial CT images captured after transcatheter arterial embolization (TAE). (A) Plain CT image captured immediately after the first TAE. (B) Plain CT image captured 1 month later. These images depict the migration of *N*-butyl-2-cyanoacrylate glue to the distal ileal artery 1 month post-TAE (arrows). (C) Contrast-enhanced CT (arterial phase) image at the same level as the plain CT image shown in (A). The *N*-butyl-2-cyanoacrylate glue was masked by the contrast medium, making it difficult to distinguish from normal vessels.

Several points should be considered in this case: (1) the anatomy of the target artery exhibited atypical features; (2) procedure was complicated and required blood flow modification using coils; (3) TAE was performed in the upper GI tract; (4) NBCA concentration was low; (5) flux of the non-target NBCA–lipiodol mixture was negligible; and (6) no apparent symptoms related to ischaemic enterocolitis were observed for >1 month postoperatively. These conditions may contribute to the development of complications and a delayed diagnosis. Particularly when the dilution rate of NBCA is low, as the distal organs would then suffer from inadequate blood flow through the collateral vessels, which may cause severe intestinal ischaemia.^1^ The mechanism underlying the metachronous events involves the migration of NBCA glue over time. It is also possible that NBCAs with lower dilution rates caused embolism migration. Therefore, attention should be paid to the NBCA dilution rate. In this case, comparisons of plain CT scans obtained at 1-month intervals post-TAE showed migration of the NBCA glue to the distal part of the ileal artery (Figure 5A and B). On contrast-enhanced CT, the NBCA glue was masked by the contrast medium, making it difficult to distinguish it from the normal vessels (Figure 5C). Thus, plain CT scans should be closely evaluated after a successful NBCA-based TAE to detect off-target embolization. The presence of arterial wall calcification caused by atherosclerosis may also confound the analysis. Therefore, a plain CT scan performed before TAE should be used as the baseline for comparison.

This case demonstrates that TAE using an NBCA–iodized oil mixture effectively treats acute GI bleeding. However, off-target embolization can occur. NBCAs with low dilution rates may induce severe complications; therefore, sufficient attention should be paid to the dilution rate of NBCA. Furthermore, regarding follow-up post-TAE with NBCA, a thorough assessment of plain CT scans throughout the follow-up period is warranted to identify unnoticed embolizations in non-target areas.

## Learning points

- Symptoms of off-target embolization that can occur when *N*-butyl-2-cyanoacrylate (NBCA) is used as an embolic agent may be mild or absent.
- Intestinal stenosis can occur >1 month after TAE using NBCA.
- Variations in the vascular anatomy, procedural complexity, and low NBCA concentrations may increase the likelihood of off-target embolization.
- Contrast-enhanced and plain CT scans should be performed after NBCA-based TAE.
